# Editorial for the Special Issue on “Nanoalloy Electrocatalysts for Electrochemical Devices”

**DOI:** 10.3390/nano12010132

**Published:** 2021-12-31

**Authors:** César A. C. Sequeira

**Affiliations:** Materials Electrochemistry Group, CeFEMA, Instituto Superior Técnico, University of Lisbon, 1049-001 Lisboa, Portugal; cesarsequeira@ist.utl.pt

Nanoscale science and technology dealing with materials synthesis, nanofabrication, nanoprobes, nanostructures, nanoelectronics, nano-optics, nanomechanics, nanodevices, nanobiotechnology, and nanomedicine is an exciting field of research and development in Europe, the United States, and other countries around the world [1,2,3,4]. Over the past two decades, considerable progress has been made with countless discoveries advancing the understanding of the nanoscale and enabling applications from advanced electronic, magnetic, and electrochemical devices, energy, and defense to life-saving medicines [5,6,7,8,9,10]. At the heart of these fields are specific complexes of solid units that possess sizes between one and hundred nanometers (1–100 nm), which we call “nanomaterials” [11,12]. These materials have a very large surface area to volume ratio when compared with bulk material and can be produced using chemical or physical methods. They are small enough to confine their electrons and produce quantum effects, thus leading to unexpected chemical, mechanical, magnetic, electronic, electrochemical, and optical properties. They are classified into three major categories—namely, nanoparticles, nanoclays, and nanoemulsions. Nanoparticles can exist as nanostructures or as composites. All nanostructures can be built from elementary units or blocks having low dimensionality. It could either be zero dimension, one dimension, two dimensions, or three dimensions. If only one dimension of a three-dimensional nanostructure is of nanoscale, the structure is referred to as a quantum well; if two dimensions are of nanometer scale, the structure is referred to as a quantum wire; and if all three dimensions are of nanometer scale, the structure is referred to as a quantum dot. Hence, a quantum dot has all three dimensions in the nanorange and is the ultimate example of nanomaterials. The word quantum is associated with these three types of nanostructures because changes in properties arise from the physics of quantum mechanics. Nanoparticles are either organic or inorganic. Inorganic nanoparticles are more stable than organic nanoparticles but are limited by their stability in terms of either mechanical or chemical properties. Nanoclays are prepared using ion-exchange reactions, and usually, their chemical composition is characterized by FTIR. Nanoemulsions can be synthesized by high (e.g., ultrasonification, microfluidization) or low energy emulsification (e.g., solvent displacement, phase inversion composition). In parallel to the development of applications, considerable worldwide attention has focused on the potential implications of nanomaterials and vastly improved our understanding of nanosafety and other emerging nanosciences. Early focus areas of nanoscience, such as nanophotonics, have grown into their own fields. New suites of tools are now available to fabricate and characterize nanomaterials and enable their integration into devices and systems. Additionally, a vibrant, interdisciplinary, and international nanotechnology community has developed [13,14,15,16,17,18,19,20,21,22,23].

With this in mind, and considering that, as a chemical engineer with a doctoral thesis in material science, I have been working for many years in applied domains of electrochemistry in which metallic, bimetallic, trimetallic and other nanoalloys find several applications in the areas of partial oxidation/reduction of fuel cells, catalysis of methane and synthetic ammonia, energy storage, water electrolyzers, electrochemical detectors, contaminant degradation in water and wastewater, electronic devices, nanomedicine, cellphones, biosensing, drug delivery, electric cars, electrochemical sensors, electrodegradation devices, hydrogen peroxide producers, etc., I was encouraged by the Nanomaterials Coordination Office to advance a Special Issue providing examples of ongoing nanotechnology research, current applications, and an overview of new elements of nanoalloy electrocatalysts, reporting their synthesis, characterization, modeling, unique properties and low cost for electrochemical devices. 

Many techniques have been developed to synthesize and fabricate nanomaterials and, in particular, metallic nanoparticles, with controlled shape, size, dimensionality, and structure. The performance of these materials depends on their properties. The properties, in turn, depend on the atomic structure, composition, microstructure, defects, and interfaces, which are controlled by thermodynamics and kinetics of the synthesis. There are two technical approaches for the synthesis of metallic nanoparticles—namely, top-down and bottom-up processes. The former involves the breaking down of the bulk material into nanosized structures or particles. It is inherently simpler and depends either on the removal or division of bulk material or on miniaturization of bulk fabrication processes to produce the desired structure with appropriate properties. Usually, the prepared surface structure contains many impurities and structural defects. Examples of top-down techniques are high-energy wet ball milling, electron beam lithography, atomic force manipulation, gas-phase condensation, aerosol spray, etc. The latter approach refers to the build-up of material from the bottom: atom by atom, molecule by molecule, or cluster by cluster. The organometallic chemical route, reversed micellar route, sol–gel synthesis, colloidal precipitation, hydrothermal synthesis, template-assisted sol–gel, electrodeposition, etc., are some of the well-known bottom-up techniques reported for the preparation of diverse nanoparticles. It has been found that the Gibbs free energy, the thermodynamic equilibrium, and the kinetic methods are the main strategies for the synthesis of nanoparticles by the bottom-up approach. 

Many of the bottom-up techniques (or molecular nanotechnology) are still under development or are just beginning to be used for commercial production, belonging to the process intensification philosophy, whose peculiar operative conditions intend to maximize the mass/heat/momentum transfer inside the processing unit itself [2,24,25]. The objective of process intensification is not so as to “increase the efficiency and reduce the plant size”, but also to enhance the production efficiency and process safety, to lower the cost, and to minimize the waste at source for reducing the environmental pollution [2]. 

The spinning disk reactor is a promising process intensification technology for heat and mass transfer rate enhancement. The hydrothermal route is another relevant technique that, according to Chen et al. [25] allowed the synthesis of well-dispersed zinc oxide and carbon co-modified LiFePO_4_ spherical particles (promising cathode materials for lithium-ion batteries). Green and bio-based technologies permit also to achieve reproducible nanomaterials applied in biomedical, medical, and electrochemical fields. In this context, electrochemical devices that address rising energy demands with the corresponding disadvantages are experiencing a change in this situation by process intensification. For example, Carmo et al. [5] reported Pt_57.5_ Cu_14.7_ Ni_5.3_ P_22.5_ bulk metallic glass (Pt-BMG) nanowires, which circumvent the performance problems of electrochemical devices. Pt-BMG nanowires catalysts were fabricated by a nanoimprinting approach, with high conductivity and activity for methanol and ethanol oxidation. After 1000 cycles, these nanowires, maintain 96% of their performance—2.4 times as much as conventional Pt/C catalysts. Their properties make them ideal candidates for energy conversion/storage and sensors. Using a contemporary PtCu-alloy electrocatalyst as a model system, a unique approach caused by the process of dealloying was presented by Ruiz-Zepeda et al. [11]. Observing detailed structures of the same nanoparticle at different stages of electrochemical treatment, they found new insights on properties such as size, faceting, strain, and porosity development. Furthermore, based on microscopy data and Kinetic Monte Carlo (KMC) simulations, they provided further feedback into the physical parameters governing electrochemically induced structural dynamics. This unique approach paves the way for an understanding of the structure–stability relationship.

In the area of alkaline polymer electrolyte fuel cells (APEFCs), a series of Ni-Cu binary alloy films were prepared using a combinatorial magnetron computing method, and their corresponding electrocatalytic performances toward HOR in alkaline media were systematically studied [19]. A volcano-type correlation between HOR activity and Cu doping content was revealed. The impart of Cu addition on the HOR catalytic activity (and antioxidation property) of Ni was deeply understood.

Allowing two kinds of metal, such as platinum group metals with 3d transition metals, has a great interest in advanced technological applications due to enhanced chemical stability and catalytic activities via increased active surface area [20,22]. In particular, loading Pt-based nanoparticles with ferromagnetic Fe, Co, and Ni atoms result in a reduction in the production cost, while the catalytic activities increase, such as hydrogen evolution and oxygen reduction reactions, the magnetic properties controlled, such as coercivity and saturation magnetization, for practical applications [6]. While Pt Fe alloy nanoparticles are widely used for permanent magnets (uniaxial magnetocrystalline anisotropy) and good chemical stability, PtNi nanoparticles is a promising material for magnetoresistive sensors, ultra-high density data recording technologies, and magnetic resonance imaging (MRI) applications, and exhibits better performance and durability for catalytic activities than commercially available Pt/C [26].

Electrodeposition is one of the practical methods to make nanocrystalline (NC) materials. There are two classes of deposition methods—electrodeposition and electroless deposition; both are inexpensive methods to manufacture relatively ductile metallic bulk materials or films/coatings. Electrodeposition is usually used for making thicker films (with higher deposition rates), while electroless deposition is typically employed for making high-quality thin coatings. The grain size and microstructure of electrodeposited films are controlled by tuning deposition parameters. Furthermore, these deposition parameters can be changed continuously or discontinuously to make functionally graded materials. The density, thickness, grain orientation of the deposited films can also be affected by the substrate conditions. To date, electrodeposition methods have been used to make various NC-materials (<100 nm in grain size) including Ni, Co, Pd, Cu, Zn, Ni-P, Ni-Fe, Ni-W, Zn-Ni, Ni-Fe-Cr, Ni-SiC, etc. The hardness, wear resistance, and electrical resistivity of most of these NCs are strongly dependent on grain size, surface morphology, and preferred orientation, and these effects are better exhibited by pulse than by direct current electrodeposition.

The corrosion behavior of NC alloys has been assessed by several techniques in various environments [27,28,29,30,31]. First results on the corrosion of crystallized melt-spun materials were obtained in Fe-Ni-Cr-P-B alloys in chloride–sulfate and sulfuric acid solutions [27,28]. It was determined that the corrosion resistance of the NC made of Fe_32_-Ni_36_-Cr_14_-P_12_-B_6_ was significantly greater than that of the amorphous counterpart. This improved corrosion resistance was attributed to the observed enrichment of Cr in the passive layer due to rapid interface boundary diffusion. Zeiger et al. [29] studied NCs made of Fe8Al in sodium sulfate solution. At the earlier stage of the formation of oxide film, this region becomes impoverished in Al. Diffusion of Al occurs due to a gradient in the Al content between the region beneath the Al oxide and the bulk alloy. The diffusion preferentially occurs through the grain boundaries and is enhanced in NC alloy due to its higher density of intergranular paths. As in NC alloy, the protective Al oxide is formed rapidly, and the uniformity and the compactness of passive film are enhanced. This film constitutes an effective barrier and protects the alloy against corrosion. Thus, it is possible to apply Fe8Al alloy films on carbon steel surfaces as protective measures. Barbucci et al. [30] investigated the corrosion behavior of Cu_90_Ni_10_ alloy in neutral media containing chlorides. They reported a decrease in the protective properties of the passive layer in the nanostructured alloy that was found to depend on the oxygen concentration. The increased amount of grain boundary in the nanostructured alloy could justify the loss of oxide compactness as a result of its irregular growth on the surface. Alves et al. [31] found that NC alloys made of (Ni_70_ Mo_30_)_90_B_10_ are less sensitive to corrosion in alkaline solutions than the coarse-grained material.

Engineering alloys for high-temperature applications rely on the formation of protective oxide films such as Al_2_O_3_ and Cr_2_O_3_ to resist high temperature and corrosive environments. The high density of grain boundaries in an NC material provides fast diffusion paths, promoting selective oxidation of protective oxide scales with better adhesion to the substrate. Consequently, the percentage of passivating elements (such as Al and Cr) in the alloy/composite that is required to form a complete protective oxide scale can be substantially reduced. Experimental results indicate that when the grain size of the NC comprising Ni-20Cr-Al coatings was ~60 nm, alloys containing ~2 wt% Al could form a complete α-Al_2_O_3_ scale at 1000 °C in air. This concentration is only one-third of the required Al% for Ni-20Cr alloys with normal grain size [8].

The interest in Ti alloys and TiAl intermetallic alloys is growing in recent years because of their excellent ratio between mechanical properties and density [32]. However, for the last 15 years, the application was restricted at temperatures up to 873–973 K by the resistance to oxidation. For this reason, it was of great importance to study protective coatings able to raise its temperature of performance above 1073 K. A TiAl_3_ layer was obtained on the surface of TiAl intermetallic samples, depositing an Al coating by arc physical vapor deposition, followed by a thermal treatment. The mechanical behavior of the thermally treated layer was investigated by means of scratch tests and nanoindentation. Oxidations at 1123 K were carried out on coated and uncoated samples, in order to study the effect of the coating on the oxidation resistance of the samples. The aluminide coating provides protection against oxidation in air at the considered temperatures due to its ability to form a continuous Al_2_ O_3_ scale on the surface. Thus, these nano- or submicro-alloy coatings provide powerful tools for TiAl intermetallics to be used as high-temperature structural materials [8].

Many of the latest contributions on the possibilities and challenges to overcome the drawbacks that nanotechnology still exhibits and that prevent its large-scale development have been collected above, emphasizing nanoalloys for electrochemical devices. This Special Issue deals with these multimetallic nanoparticles and their theoretical and computational studies, as well as their synthesis, characterization, chemical and physical properties, and recent electrochemical application-driven research. The Guest Editor is very satisfied with the contributions received and finally published.

## Data Availability

Not applicable.
